# Combination of Nanomicellar Technology and In Situ Gelling Polymer as Ocular Drug Delivery System (ODDS) for Cyclosporine-A

**DOI:** 10.3390/pharmaceutics13020192

**Published:** 2021-02-01

**Authors:** Eleonora Terreni, Erica Zucchetti, Silvia Tampucci, Susi Burgalassi, Daniela Monti, Patrizia Chetoni

**Affiliations:** 1 Department of Pharmacy, University of Pisa, Via Bonanno 33, 56126 Pisa, Italy; eleonora.terreni@farm.unipi.it (E.T.); erica.zucchetti@phd.unipi.it (E.Z.); susi.burgalassi@unipi.it (S.B.); daniela.monti@unipi.it (D.M.); patrizia.chetoni@unipi.it (P.C.); 2 Centro 3R (Inter-University Center for the Promotion of the 3Rs Principles in Teaching & Research), 56122 Pisa, Italy

**Keywords:** nanomicelles, in situ gelling polymer, ocular, pharmacokinetics in rabbits, Cyclosporine-A

## Abstract

A combination of in situ gelling systems and a loaded drug self-assembling nanomicellar carrier was chosen in this study as a new potential Ocular Drug Delivery System (ODDS) for Cyclosporine-A (CyA), a poorly water-soluble drug. Two non-ionic surfactants (d-α-tocopherol polyethylene glycol succinate, VitE-TPGS and polyoxyl 40 hydrogenated castor oil, RH-40) were used to produce the nanomicelles. The physical–chemical characterization of the nanomicelles in terms of CyA entrapment (EE%) and loading efficiency (LE%), cloud point (CP), regeneration time (RT), size and polydispersity index (PI) allowed us to select the best combination of surfactant mixture, which showed appropriate stability, high CyA-EE (99.07%), very small and homogeneous dimensions and favored the solubilization of an amount of CyA (0.144% *w*/*w*) comparable to that contained in marketed emulsion Ikervis^®^. The selected nanomicellar formulation incorporated into optimized ion-sensitive polymeric dispersions of gellan gum (GG-LA: 0.10, 0.15 and 0.20% *w*/*w*) able to trigger the sol–gel transition after instillation was characterized from technological (osmolality, pH, gelling capacity, rheological behavior, wettability, TEM and storage stability at 4 and 20 °C) and biopharmaceutical points of view. This new combined approach allowed us to obtain clear aqueous dispersions that were easy to instill and able to form a viscous gel when in contact with the tear fluid, improving CyA ocular bioavailability. Furthermore, this new ODDS prevented CyA transcorneal permeation, exhibited low cytotoxicity and prolonged the CyA resident time in the precorneal area compared to Ikervis^®^.

## 1. Introduction

The design of Ocular Drug Delivery Systems (ODDS) represents a real challenge for pharmaceutical researchers given the complexity of the very specialized structure of the eye. The eye plays a fundamental role in the quality of human life and is able to preserve all its peculiar visual functions by arranging defensive mechanisms that involve anatomical structures (stratified corneal epithelium, stroma and sclera), physiological factors (tear turnover, blinking, choroidal or conjunctival blood flow and lymphatic clearance) and metabolic components (efflux pumps/enzymes). For these reasons, it is not simple for exogenous substances such as drugs, generally administered as conventional, well-accepted eye drops, to reach the target site into the eye, although it can be affected by serious and disabling pathologies [1,2]. The main criteria for the development of several ODDS are the capability of the systems to prolong corneal contact time and to improve corneal penetration of the drug molecules [1,2,3,4,5,6]. Moreover, the design of ODDS containing drugs with very low water solubility (~23 μg/mL), high molecular weight (1202.6 Da) and high lipophilicity, such as Cyclosporine-A, constitutes a further problem [3,6,7,8]. In this case, the ODDS should both deliver the drug in the solubilized form to corneal surface and overcome the disadvantages of the limited drug retention time in the pre-corneal area and the sometimes scarce drug transcorneal penetration. Considerable attention has been paid to nanocarrier formulations, as they seem to favor a significant improvement in ocular bioavailability of Cyclosporine-A, partially solving the problem of its poor water solubility. Nanoemulsions [9,10], niosomes and liposomes [11], polymeric and lipid nanoparticles [12,13,14,15] and micelles [16,17,18,19,20] represent the main attempts to develop effective ocular delivery systems. Some of them have achieved commercial development: the first FDA-approved product with 0.05% *w*/*w* of CyA was marketed in 2003 and was an oil-in-water nanoemulsion (Restasis^®^). Since then, few other products have been marketed, up to the recently approved 0.09% *w*/*w* Cyclosporine-A nanomicellar system (Cequa^®^) [21]. Generally, nanocarriers provide a slow drug release and a relatively longer drug retention time at the corneal surface, and the presence of surfactant or cosolvent promotes an enhancer activity, thus improving the drug corneal permeation and the therapeutic effectiveness. The further addition of ingredients to nanocarrier formulations has been reported as an interesting strategy; widely used polymeric materials and in situ gel-forming systems appear to be particularly interesting and promising. They are characterized by low viscosity values before the instillation in the eye, but once administered, their viscoelasticity increases for the formation of covalent (chemical cross-linking) or non-covalent bonds (physical cross-linking) between the polymeric chains. The production of the gel structure is rapidly triggered in response to biological condition changes such as temperature, pH and presence of specific ions. The production of the gels often determines a release of the drug to the ocular structures in a sustained or controlled manner. Such a type of product consisting of the well-known Gelrite^®^ was marketed in the early 1980s (Timoptic-XE^®^, Merck and Co. Inc., Whitehouse Station, NJ, USA) and subsequently proposed by numerous researchers and pharmaceutical companies. Different types of polymers (gellan gum, hydroxyethylcellulose, poloxamer, poly-*N*-isopropylacrylamide-hyaluronic acid copolymers, chitosan derivatives and polyacrilic acid) have been used for the development of several in situ gelling systems containing free drugs or drugs loaded into microemulsions and nanoparticles [22,23] or in micelles based on non-ionic surfactants (d-α-tocopheryl polyethylene glycolsuccinate). Thermosensitive di-block polymers such as PLGA or PCL were also coupled with methoxy polyethylene glycol, able to undergo a sol-to-gel transition and form micelles for the ocular delivery of CyA. Although it has been proven that they are more effective in increasing the bioavailability and/or reducing the ocular toxicity of the drug, blurred vision due to excessive viscosity was reported as an adverse effect [24]. This unpleasant effect, although to a lesser extent than those observed for ointments, is associated with other positive features such as decreased drug nasolacrimal drainage, reduced systemic side effects and possibility to administer a well-determined and reproducible amount of drug, in contrast to the typical gel [25].

Therefore, a combination of two different strategies, in situ gelling systems and a drug-loaded nanomicellar carrier, was chosen in this study, with the perspective of the development of a new potential platform for ocular delivery of poorly water-soluble active compounds (Cyclosporine-A). Indeed, to date, such a type of ODDS has not yet been developed for the delivery of CyA. In particular, in the current research, nanomicellar formulations containing different molar ratios of two non-ionic surfactants (d-α-tocopherol polyethylene glycol succinate, VitE-TPGS and polyoxyl 40 hydrogenated castor oil, RH-40) were prepared and characterized in terms of drug entrapment efficiency (EE%), loading efficiency (LE%), cloud point (CP), regeneration time (RT), particle size, polydispersity index (PI) and other technological parameters. The best combination of the mixture of the two surfactants was selected to prepare the nanomicellar formulation to be incorporated into ion-sensitive polymeric dispersions of gellan gum, which was chosen as it forms a stable viscous hydrogel when in contact with the tear fluid due to the appropriate balance between the divalent cations in both the formulation and the tear fluid. It was hypothesized that even a slight increase in viscosity typical of a pathological situation such as Dry Eye Syndrome (DES), where the concentration of tears is poor, could significantly increase the drug residence time in the precorneal area, promoting a greater therapeutic efficacy of the drug. Since the osmolality of the tear fluid often increases in DES due to the excessive evaporation of the tear fluid and/or inadequate fluid secretion [24] determining higher ion concentrations, this approach to the development of an ion-sensitive gelling system could be a winning strategy.

In order to obtain fluid gels, three different aqueous isotonic dispersions of gellan gum (GG-LA = 0.10, 0.15 and 0.20% *w*/*w*) were prepared in the presence of the monovalent (Na^+^ and K^+^) and divalent cations (Ca^2+^ and Mg^2+^). The formulations were characterized from a technological point of view to verify the formation of nanomicelles into the gelling system and their suitability for ocular application (contact angle). Rheological studies were performed to characterize both the viscous and elastic moduli and gelling capacity of the combined system in the presence of nanomicelles. Moreover, a short-term chemical stability was carried out at room temperature and 4 °C. Finally, both the in vitro permeation of CyA released by the new ODDSs was evaluated through reconstituted corneal tissues and a pharmacokinetic study on the retention time of CyA in the rabbit tear fluid was performed, choosing the marketed Ikervis^®^ as reference.

## 2. Materials and Methods 

### 2.1. Chemicals

The following materials were used as received: Cyclosporine A (CyA, Poli Industria Chimica S.p.A, Milan, Italy); d-α-Tocopherol polyethylene glycol succinate (VitE-TPGS, Kolliphor^®^TPGS) and Polyoxyl 40 Hydrogenated Castor Oil (RH-40, Kolliphor^®^RH-40), both provided by BASF, (Ludwigshafen, Germany); Gellan Gum (GG-LA, Kelcogel CG-LA), kindly provided by CP-Kelco, (Atlanta, GA, USA).

All other chemicals and solvents were of analytical grade. Ultrapure water was obtained using MilliQ^®^ plus apparatus (Millipore, Milan, Italy).

### 2.2. Cell Cultures, Reconstituted Tissues and Animals

The rabbit corneal epithelial cell line (RCE, European Cell Culture Collection, N° 95081046, ECACC, Salisbury, UK) was used for the cytotoxicity test. The growth medium had the following composition: Dulbecco’s modified Eagle’s medium (DMEM) with Ham’s nutrient mixture F12 (1:1) added of l-glutamine (1% *v*/*v*), penicillin (100 IU/mL), streptomycin (0.1 mg/mL), amphotericin B (0.25 μg/mL), fetal bovine serum (15% *v*/*v*) (Gibco Invitrogen S.r.l., Milan, Italy), epidermal growth factor (10 ng/mL) and insulin (5 mg/mL) (Sigma Chemical Co., St. Louis, MO, USA).

COR-100 EpiCorneal™ reconstituted corneal tissues were obtained from MatTek Corporation (Ashland, MA, USA) and were used as biological substrate to evaluate the capability of CyA to permeate through the corneal tissues following the protocol proposed by MatTek.

Male New Zealand albino rabbits weighing 2.8–3.5 kg were purchased from Pampaloni Rabbit (Pisa, Italy). They were housed in standard cages in a light-controlled room (10 h dark/14 h light cycle) at 19 ± 1 °C and 50 ± 5% relative humidity and were given a standard pellet diet and water ad libitum. During the experiments, the rabbits were placed in restraining boxes to which they had been habituated, and they were allowed to move their heads and eyes freely [26]. For in vivo studies, the rabbits were used and treated according to the “Guide for the Care and Use of Laboratory Animals”. All experimental procedures were carried out in accordance with the ARVO Statement for the Use of Animals in Ophthalmic and Vision Research and the European Union guidelines for the use of animals in research, and were approved by the Ethical and Scientific Committee of the University of Pisa and by the Italian Minister of Health (n. 486/2020-PR, 18 May 2020). The experimental treatments were carried out under veterinary supervision.

### 2.3. Preparation of Cyclosporine-A Loaded Nanomicellar Formulations NxCyA-ASN

Cyclosporine-A loaded Assembling Surfactant Nanomicelles (Nx/CyA-ASN) were prepared by using a binary mixture of two non-ionic surfactants, VitE-TPGS and RH-40, at three different molar ratios following the already reported procedure [27].

Briefly, an appropriate amount of VitE-TPGS was first melted at 50 °C in a closed vial for one hour to promote fusion by later adding different amounts of RH-40, while maintaining VitE-TPGS:RH-40 molar ratios of 2.25:1, 1:1 and 0.5:1 for N1, N2 and N3CyA-ASN, respectively, as reported in Table 1. Then, CyA (0.15% *w*/*w*) and water as solvent were added to the blend at the same temperature. The homogeneous final mixture was stirred overnight at room temperature and finally filtered through filters (0.22 μm pore size, Minisart NML^®^ cellulose acetate filters (Sartorius AG, Göttingen, Germany)) to remove aggregates and other foreign particulates. 

### 2.4. Preparation of In-Situ Gelling Systems (Fx)

Three aqueous polymeric dispersions (F1–F3) containing different amounts of GG-LA corresponding to 0.10, 0.15 and 0.20% *w*/*w*, respectively, were added in appropriate amounts of inorganic salts and of mannitol as isotonizing agents. The NaH_2_PO_4_·H_2_O (0.3% *w*/*w*) and Na_2_HPO_4_ (0.17% *w*/*w*) salts were solubilized in ultrapure water at 80 °C containing mannitol (0.05% *w*/*w*), EDTA sodium salt (0.01% *w*/*w*) and MgCl_2_ (0.01% *w*/*w*). Then, GG-LA was introduced at the same temperature to favor its dissolution. The final dispersions were stirred for 1.0 h up to complete hydration of the polymer, replenishing the evaporated water at the end of the solubilizing process.

### 2.5. Preparation of Cyclosporine-A Loaded Assembling Surfactant Nanomicelles In-Situ Gelling Systems (Fx/N2CyA-ASNg)

**Scheme 1:**
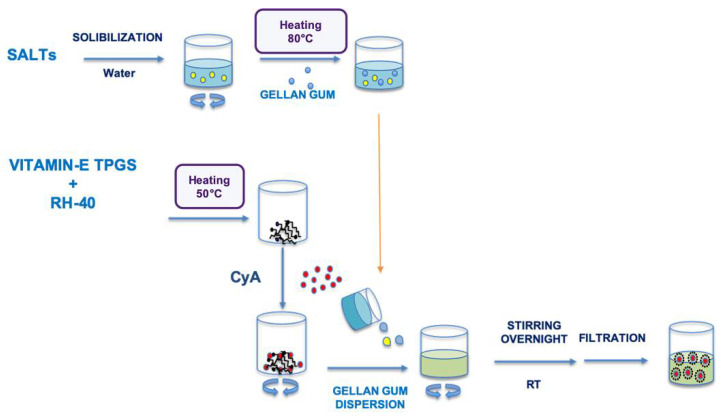
Graphical representation of the different steps to obtain the Fx/NxCyA-ASNg formulations.

The Assembling Surfactant Nanomicelles in-situ Gelling Systems loaded with CyA (Fx/N2CyA-ASNg) were prepared exclusively with the 1:1 molar ratio surfactant mixture (N_2_, binary mixture, corresponding to 0.6% *w*/*w* of VitE-TPGS and 1.0% *w*/*w* of RH-40), following the method already described in Section 2.3 and substituting the amount of water with the same amount of each polymeric dispersion (F1–F3). CyA (0.15% *w*/*w*) was introduced in all formulations, and the final mixtures were stirred overnight at room temperature and purified by filtration through 0.22 μm pore size filters to remove impurities and to obtain a sterilized preparation. A schematic representation of the Fx/N2CyA-ASNg system is represented in Scheme 1. 

### 2.6. Determination of Critical Micellar Concentration of VitE-TPGS:RH-40 (1:1) Binary Mixture

Experimental critical micellar concentration (CMC) of the VitE-TPGS:RH-40 binary mixture with 1:1 molar ratio (N2), and of the single surfactants (VitE-TPGS and RH-40), was determined by applying the partially modified method proposed by Cholkar et al. (2014) with iodine as probe [28].

The aqueous dispersions of the single surfactants (VitE-TPGS and RH-40), or of the blend of two in 1:1 molar ratio, were diluted with ultrapure water to obtain a surfactant concentration ranging between 0.0001 and 1% *w/w*. Twenty-five microliters of an iodine solution containing tri-iodide ion (I_3_^−^, 40.0 mM KI_3_), prepared by dissolving appropriate amounts of iodine and potassium iodide in ultrapure water, was added to 5.0 mL of each dilution of the different surfactant aqueous dispersions. The resulting solutions were incubated at room temperature for 15 h under darkness conditions, and the absorbance was measured at 366 nm (UV-2101PC spectrophotometer, Shimadzu, Italy) to evaluate the shift from 460 to 366 nm due to the iodine partition into the hydrophobic core of nanomicelles. The measured absorbance values were plotted against the logarithm of the concentration of each sample (% *w/w*), and the value of CMC was taken by the coordinates of the point of intersection of regression straight lines calculated on the experimental points. The test was repeated three times on three different diluted samples for each single surfactant and for the blend of VitE-TPGS and RH-40.

Furthermore, theoretical critical micellar concentration (CMC) of the surfactant mixture was calculated by using the following equation [19,29,30]:

$$\frac{1}{\mathrm{CMCmix}}=\frac{\mathrm{XTPGS}}{\mathrm{CMC}\;\mathrm{TPGS}}+\;\frac{\mathrm{XRH}-40}{\mathrm{CMC}\;\mathrm{RH}-40}$$

where CMCmix is the theoretical CMC of micelles based on the two-surfactant mixture, XTPGS and XRH-40 are the molar fractions of VitE-TPGS and RH-40, and CMC RH-40 and CMC TPGS are the experimental CMC detected for the two surfactants.

### 2.7. Characterization of NxCyA-ASN 

#### 2.7.1. Dynamic Light Scattering Analysis

The average hydrodynamic diameter (D_h_) and polydispersity index (PI) of the NxCyA-ASNs were determined by measuring the rate of fluctuations in laser light intensity scattered by the nanomicellar formulations immediately after their preparations by using a Dynamic Light Scattering (DLS) analysis (Beckman Coulter^®^ N4 Plus, Beckman Coulter s.r.l, Milan, Italy). Five minutes prior to light-scattering measurements, each sample was diluted with ultrapure water previously filtered through a 0.45 μm pore size filter (polyethersulfone membrane, Millipore Express PLUS, Merck, Italy). The final concentration was chosen on the basis of the measurement intensity ranging from 5 × 10^4^ to 1 × 10^6^ counts per second (cps). The average hydrodynamic diameter (D_h_) for each NxCyA-ASN formulation was obtained on three different samples for which 6 runs were carried out using an angle of 90° [31] and run time of 200 s at 20 °C.

#### 2.7.2. Determination of the Amount of Solubilized CyA (CyA-In), CyA Entrapped (CyA-EE) and CyA Loading Efficiency (CyA-LE) of NxCyA-ASN Formulations

The amount of CyA solubilized (CyA-In) by using the different binary surfactant mixtures was determined by RP-HPLC [25]. An aliquot (1.0 mL) of each NxCyA-ASN was added to acetonitrile (ACN, 20.0 mL) to favor the dissolution of CyA into organic solvent [32]. The amount of CyA was detected by HPLC analysis, and each analysis was repeated on three different samples.

The experimental results on the amount of CyA solubilized were used to calculate the percent of CyA entrapment (CyA-EE) and the loading efficiency (CyA-LE) of nanomicelles prepared with the different ratios of surfactants according to Equations (1) and (2), respectively.

CyA-EE (% *w*/*w*) = (mass of CyA in nanomicelles × 100)/(mass of CyA added in the mixture) (1)

CyA-LE (% *w*/*w*) = (mass of CyA in nanomicelles × 100)/(mass of CyA added + mass of the other excipients) (2)

#### 2.7.3. Determination of Thermal Stability and Regeneration Time (RT) 

The stability of NxCyA-ASN nanomicelles after heating was preliminary determined by visually observation, followed by turbidimetric analysis according to Terreni et al. 2020 [27]. Briefly, aliquots (2.0 mL) of the different nanomicellar preparations (N1–N3/CyA-ASN) were placed into closed glass vials and heated up to 80 °C by using a water bath. During the experiment, the samples were continuously observed, and their absorbance at 400 and 500 nm (Spectrophotometer UV-2101PC, Shimadzu, Italy) was measured. The temperature at which the nanomicellar preparation became visually turbid or milky-white and their absorbance values were detected [33] and indicated the cloud point (CP). The CP for NxCyA-ASN formulations was determined at least in triplicate. 

Afterwards, the regeneration time (RT) for the N1–N3/CyA-ASN preparations was visually monitored while cooling down the nanomicellar preparations to room temperature. The regeneration time is defined as the time necessary to transform the nanomicellar cloud dispersions into the native clear dispersions with the same clarity observed before heating. 

### 2.8. Characterization of Fx/N2CyA-ASNg 

#### 2.8.1. Solubilized CyA (CyA-In), CyA Entrapment (CyA-EE) and CyA Loading Efficiency (CyA-LE), Dh, PI, CP, RT for Fx/N2CyA-ASNg Formulations

The amount of solubilized Cyclosporine-A into the in situ gel forming nanomicellar systems was determined by RP-HPLC after dilution of an aliquot (1.0 mL) of Fx/N2CyA-ASNg formulations with acetonitrile (ACN, 20.0 mL) to promote drug dissolution and polymer precipitation. After centrifugation at 13,000 rpm for 10 min (Micro CL 17, Thermo Fisher, Milan, Italy), the supernatant was analyzed by HPLC to quantify the drug. Each analysis was repeated on three different samples. Data were used to calculate percentage of CyA solubilized (CyA-In), CyA entrapped (CyA-EE) and CyA loaded (CyA-LE) according to Equations (1) and (2) (see Section 2.7.2). Furthermore, the average hydrodynamic diameter (D_h_), polydispersity index (PI), cloud point (CP) and regeneration time (RT) were determined as previously described (Section 2.7.3).

#### 2.8.2. Osmolality and pH of Fx/N2CyA-ASNg Formulation

Osmolality and pH of the Fx/N2CyA-ASNg formulations were determined by a freezing point depression using the Osmometer (5R version, Roebling, Germany) and a digital pH-meter (611 model, Orion Research, Jacksonville, FL, USA), respectively. 

Three different batches of each formulation were produced to verify the reproducibility of the manufacturing method.

#### 2.8.3. Gelling Capacity and Rheological Analysis 

The gelling capacity (GC), defined as the time required to produce an appreciable visual modification of the viscosity of a formulation, was evaluated for Fx/N2CyA-ASNg before and after mixing with Artificial Tear Fluid (ATF, pH = 7.4) [34,35]. GC was determined by slowly adding an aliquot of each formulation to appropriate amounts of ATF maintained under magnetic stirring to simulate the biological condition of the eye during the instillation of eye drops into the conjunctival sac (volumetric ratio of 30:7). The ATF contained the typical cationic species of the human tears, and its composition was NaCl 6.8 g/L, NaHCO_3_ 2.2 g/L and CaCl_2_·H_2_O 0.084 g/L e KCl 1.4 g/L. The grade of gelation was visually graded according to the scale indicated in Table 2.

Furthermore, the rheological behavior of the colloidal dispersions was determined at 32 °C by a Haake Rheostress RS 1 (Thermo Fisher Scientific, Karlsruhe, Germany), equipped with coaxial cylinders (Z40 and Z41) at a shear rates (D) ranging from 0 to 200 s^−1^. The viscosity flow curves were recorded and analyzed by using Haake RheoWin™ Software (Version 4.61, Thermo Fisher Scientific, Karlsruhe, Germany) and the Ostwald-de-Waele law: τ = η D^N^ for Equation (3) [36,37].

D = 1.0 s^−1^ (3)

This test was performed immediately after preparation of the formulations and after the gelling process took place by addition of an adequate amount of ATF, following the experimental condition previously described. All the measurements were performed in triplicate. 

#### 2.8.4. Dynamic Viscoelastic Experiments

The viscoelastic characterization of the formulations (Fx, Fx/N2CyA-ASNg and Fx/N2CyA-ASNg/ATF) was carried out by means of a controlled-stress rheometer (Haake Rheostress RS 1, Thermo Fisher Scientific, Karlsruhe, Germany) equipped with coaxial cylinder sensor system (Z40 and Z41) using the Haake RheoWin™ Software (Version 4.61, Thermo Fisher Scientific, Karlsruhe, Germany) for data acquisition. Prior to analysis, the system was equilibrated with a five-minute waiting time to reach the selected temperature (32 ± 0.2 °C). Steady shear measurements were performed at shear rates ranging from 0 to 120 s^−1^. The analyses were performed in triplicate [38].

Oscillatory measurements were performed in two steps:

- Stress sweep analysis, in which the stress (τ) was varied between 0.1 and 100 Pa, while the frequency was kept at 5Hz to determine the linear viscoelastic region (LVR) and consequently the maximum deformation reachable by the sample;
- Frequency sweep analysis to determine the storage (or elastic) modulus (G’) and loss (or viscous) modulus (G’’), and the phase angle (δ) as a function of the frequency (1–10 Hz corresponding to 6.28 to 62.83 rad/s) at a constant stress (5 Pa) within the LVR.

#### 2.8.5. TEM Analysis 

The morphological features (size and shape) of the nanomicelles in Fx/N2CyA-ASNg/ATF gelled mixtures were observed by TEM (JEOL 100 SX, model AMT XR80B, JEOL, Tokyo, Japan) equipped with a CCD camera. The samples were prepared using the following procedure: first, 10 μL of each sample was placed onto a copper grid with a formvar/carbon (200 mesh) membrane and left for 3 min, and after removing excess solution with filter papers, the copper grid was gently washed with drops of deionized water. Subsequently, the copper grid was negatively stained with 20 μL of uranyl acetate solution (2%, *w*/*v*) for 30 s. The excess solution was removed with filter papers, and the sample was left to dry by air for 20 min. Then, it was examined under TEM, operating at an acceleration voltage of 100 kV [32,39].

#### 2.8.6. Wettability of the Nanomicellar Formulations 

Static contact angle measurements were performed on N2/CyA-ASN and Fx/N2CyA-ASNg formulations before and after the gelling process (Fx/N2CyA-ASNg/ATF) using an optical contact angle-measuring instrument (OCA 15, DataPhysics Instrument GmbH, Filderstadt, Germany). The system consisted of a high-resolution CCD video camera and a six-fold power zoom lens with integrated fine focusing; the images were recorded and analyzed by SCA 20 software. The sessile drop method was applied as follows: a volume of 5.0 μL of each formulation was dropped onto a glass slide at a rate of 50 μL min^−1^. When the spreading droplet achieved an equilibrium state, the image was taken and analyzed by the Laplace–Young fit method. Six measurements were made for each formulation [37].

#### 2.8.7. Storage Stability

The physical and chemical stability of the Fx/N2CyA-ASNg formulations was monitored over 60 days after storage of the preparations in refrigerated conditions (4 °C) and at room temperature (20 °C). 

Briefly, the formulations, once prepared, were filtered through a 0.22 μm filter under a laminar flow and packaged into different crimp sterile vials. At appropriate time intervals, the samples were collected and the concentration of CyA in the samples was determined by HPLC after appropriate dilution with ACN to calculate CyA-In% *w/w*. The amount of CyA detected in the samples was reported as a percentage of the initial amount, and the coefficient of variation (CV%) was calculated according to the equation:

(SD/mean) * 100, where SD represents standard deviation. Furthermore, the physical features (size and size distribution) of nanomicelles were evaluated by DLS to verify the effective nanostructure maintenance during the storage at the different temperature conditions [27,30]. All the measurements were repeated on three different batches for each formulation. 

#### 2.8.8. Cytotoxicity Assay

A cytotoxicity test was performed on a rabbit corneal epithelial cell line using ready-to-use cell proliferation reagent WST-1 [40]. The RCE cells were plated at 3 × 10^5^ cells/mL, in 96-well microtiter plates and treated with appropriate dilutions of N2/CyA-ASN and Fx/N2CyA-ASNg. A blank nanomicellar formulation (N2/ASN) based on the selected surfactant mixture (VitE-TPGS:RH-40, 1:1 molar ratio) and prepared following the method reported in the Section 2.3, was chosen as reference. In any case, the final surfactant concentration was in the range between 0.00016 and 0.16%. 

All the preparations were suitably diluted in growth medium up to the tested concentrations. After 15 min of exposure, the formulations were removed, the cells were washed twice with DMEM/F12 and 100 μL fresh growth medium and 10 μL reagent WST-1 was added in each well. The cells were incubated for 2 h at 37 °C, then the microplate was thoroughly shaken for 30 s and the absorbance was measured at 450 nm using the microplate reader (Asys UVM 340, Biochrom Ltd., Cambridge, UK). 

The results were expressed as percentage absorbance of the treated vs. untreated control wells according to Equation (4):

(4) $$\mathrm{Cell}\;\mathrm{viability}\%\;=\;\frac{\mathrm{Treated}\;\mathrm{Abs}\;}{\mathrm{Control}\;\mathrm{Abs}}\;\times \;100$$

#### 2.8.9. In Vitro Permeation Studies of CyA through Ocular Reconstituted Tissue

COR-100 EpiCorneal™ reconstituted tissues were chosen as biological substrate to evaluate the capability of CyA loaded into ASN formulations to permeate through corneal tissues [25]. COR-100 EpiCorneal™ reconstituted tissues were preliminary equilibrated overnight in culture medium (COR-100 ASY medium) at Standard Cell Culture Conditions (SCC, 37 °C, 5% CO_2_) in line with the Mattek protocol, whereupon the tissues were transferred into two different 12-well plates and equilibrated with 500 µL of Krebs Ringer Bicarbonate buffer (KRB, pH 7.4, receiving phase) for 30 min before the beginning of each treatment. Then, aliquots of about 100 µL of the formulations (donor phase) were applied on the tissue surface (apical layer). The commercial eye drop Ikervis^®^ (Reference, Santen Oy, Tampere, Finland) containing 0.1% *w*/*w* of CyA was used as reference. In any case, different volumes for the different formulations (tested and reference) were used to obtain the same CyA concentration at the beginning of the experiments. The plates were incubated at 37 °C. 5% CO_2_ on a horizontal plate shaker (Incubating Microplate Shaker, VWR^®^, Philadelphia, PA, USA) that determined a slight agitation to assure sink conditions. At predetermined time intervals (0.5, 1, 2, 3 and 4 h), 300 µL of KRB receiving medium was withdrawn to analyze CyA content by HPLC and replaced with the same volume of fresh medium. The experiment was performed in triplicate.

Moreover, the EpiCorneal™ tissue resistance was measured to evaluate the integrity of the corneal barrier. The measurements were performed at the beginning and end of each permeation experiment. The initial resistance (t = 0 h; TEER-1) was measured after the preliminary treatment, immediately after filling the wells with 300 (donor phase) and 500 µL (receiving phase) of KRB buffer solution using an epithelial volt-ohm meter EVOM and the EndOhm-12 chamber (World Precision, Sarasota, FL, USA). Furthermore, the resistance of the tissues was measured for each sample at the end of the permeation study (t = 4 h; TEER-2) in the same conditions. The resistance was detected on three different samples of EpiCorneal^TM^ tissues.

#### 2.8.10. In Vivo Pharmacokinetic Studies: CyA Content in Tear Fluid of Rabbits

Fifty microliters of the selected nanomicellar formulation (N2/CyA-ASN) and the same volume of the same nanomicellar preparation but added to different amounts of gellan gum (Fx/N2CyA-ASNg) were instilled into conjunctival sacs of the rabbit eyes. Then, tear fluid samples were taken from the lower marginal tear strip, using a 1.0 μL disposable glass capillary (Microcaps, Drummond Scientific, Broomall, PA, USA) at 1, 3, 5, 10, 20 and 30 min after instillation [41]. The collected sample was then put into microtube Eppendorf^®^, and the capillary rinsed with ultrapure water (1.0 μL). The resulting sample was further diluted with 98 μL of ACN, obtaining a final sample volume of 100 μL. After centrifugation at 13,000 rpm for 5 min (Micro CL 17, Thermo Fisher, Italy), the supernatant samples were analyzed by HPLC, and the amount of CyA was calculated by comparison with an appropriate external calibration curve. A commercial 0.1% *w*/*v* CyA emulsion (Ikervis^®^) was used as control.

The pharmacokinetics parameters included CyA concentration at different times reported as mean ± standard error (S.E.), the apparent first order elimination rate constants (K_e_, min^−1^) of CyA from the tear fluid and the corresponding half-lives (t_½_ = 0.693/K_e_). These parameters were calculated from the linear phase of log tear fluid concentration vs. time plots [42]. Furthermore, the CyA bioavailability was quantified as the area under curve (AUC) and was obtained by applying the linear trapezoidal rule. The AUC was calculated from 1 to 30 min after instillation of Ikervis^®^, N2CyA-ASNg nanomicellar dispersion and F1/N2CyA-ASNg nanomicellar in situ gelling systems with a lower GG-LA concentration (0.1% *w*/*w*), whereas a comparison among all the tested formulations was performed using the AUC values calculated from 3 to 30 min after ocular administration.

Statistical comparison of the tear fluid CyA concentration detected at the different times was performed using a Student’s two-tailored unpaired t-test (Prism 8 software), and statistical significance was set at *p* < 0.05.

### 2.9. HPLC Analytical Method

The quantitative determination of CyA was carried out by the reverse phase HPLC method (RP-HPLC) according to Terreni et al. (2020) [27]. Briefly, the apparatus consisted of a Shimadzu LC-20AD system with a UV SPD-10A detector equipped with an autosampler CBM-20A and a computer integrating system (Shimadzu Italia s.r.l., Milan, Italy). The injection valve was a Rheodyne with a capacity of 20 μL, and the column was a Gemini-C18 (250 × 4.6 mm, Phenomenex, Torrance, CA, USA) with a porosity of 110 Å and a particle size of 5 μm. The mobile phase (acetonitrile:water:methyl tert-butyl ether, 52:43:5 mixture containing the 0.1% *v*/*v* of 85% phosphoric acid) was heated at 80 °C and delivered at a flow rate of 1.0 mL/min. CyA detection wavelength and retention time was 210 nm and 32 min, respectively. The amount of drug in the samples was determined by comparison with appropriate external standard curves [25]. The calibration curves were obtained by applying the least square linear regression analysis by using Prism 8 software (GraphPad Software 164 Inc., San Diego, CA, USA).

For in vitro studies, the calibration curves were obtained analyzing standard dilutions, with ACN or KBR, of a CyA stock solution in acetonitrile (Solution A). 

For in vivo study, the Solution A was progressively diluted with the appropriate amount of the same solvent (ACN) to produce a series of standard solutions. Then, 98 μL of each standard solution was added to rabbit rinsed tear fluid (2 μL), centrifuged at 13,000 rpm for 5 min and 20 μL of supernatant was analyzed by HPLC. 

## 3. Results

### 3.1. Characterization of NxCyA-ASNs 

A huge number of researchers have dealt with nanomicellar formulations for ocular drug delivery in the last decade. This interest is due to their evident advantages, such as the ability to improve the aqueous solubility of hydrophobic drugs, to increase drug ocular residence time and to favor higher permeation and absorption into the ocular tissues of encapsulated drugs. The efficacy of a nanomicellar formulation greatly depends upon the components (surfactants, polymers and drugs) chosen for their assembly, as well as on the technological features of the nanomicelles (structure and size). 

In our study, three different nanomicellar formulations were developed, and the ability of the surfactant mixtures to self-assemble forming suitable formulations in terms of appropriate shape, homogeneous size, absence of aggregates and optimized amount of encapsulated Cyclosporine-A was evaluated. The nanomicelles were obtained using a binary composition of non-ionic surfactants, namely VitE-TPGS and RH-40, mixed at different selected molar ratios. The technological evaluation of the different nanomicellar formulations produced the results summarized in Table 3. The change in the molar ratio between the two surfactants determined substantial differences in the characteristics of the self-assembling nanomicelles. In fact, the reduction in VitE-TPGS: RH-40 molar ratio from 2.25:1 to 1:1 in the nanomicellar formulations allowed us to maintain a homogeneous very small size with D_h_ values of 15.70 and 15.80 nm and narrow PI intervals (0.26 and 0.16) for N1CyA-ASN and N2CyA-ASN, respectively. A further decrease in VitE-TPGS:RH-40 to molar ratio 0.5:1, corresponding to the concentration of 0.3% *w*/*w* VitE-TPGS in the N3CyA-ASN, determined a self-assembly of less homogeneous nanomicelles with higher D_h_ (16.90 ± 0.28 nm) and PI (0.38 ± 0.03), suggesting a poor self-structuring capability of the surfactants in a stable nanomicellar system. Moreover, the CyA solubilization degree was extremely low for N3CyA-ASN and equal to 0.063% *w*/*w*, which was well correlated with low CyA-EE (55.86 ± 2.21% *w*/*w*) and CyA-LE (2.55 ± 0.10% *w*/*w*) values. More interesting data were obtained for the N1CyA-ASN and N2CyA-ASN nanomicellar formulations, which showed higher CyA-EE and CyA-LE values, ranging from 92.83 ± 0.83% *w*/*w* to 99.07 ± 1.61% *w*/*w* and from 5.12 ± 0.25 to 8.22 ± 0.14% *w*/*w*, respectively. 

The optimal molar ratio between VitE-TPGS and RH-40 was 1:1, which led to the solubilization of 0.144% *w*/*w* CyA, comparable to the amount present in the European marketed emulsion Ikervis^®^ (0.1% *w*/*w* CyA, Santen Oy, Tampere, Finland) by exploiting nanomicellar technology. 

The cloud point (CP) was another physicochemical parameter measured for NxCyA-ASN: all the developed nanomicellar formulations visually appeared as clear solutions at room temperature, and no significant absorbance values were recorded at 400 and 500 nm. When the formulations were heated, the absorbance values increased, allowing us to define CP values of 45 and 40 °C for the formulations N2CyA-ASN and N3CyA-ASN, respectively. On the contrary, no absorbance values were recorded up to 80 °C (limit of the experiment) for the formulation N1CyA-ASN. These results suggested that a greater amount of VitE-TPGS in N1CyA-ASN (1.35% *w*/*w*) could improve the thermal resistance of the nanomicelles. In general, heating over the CP temperature determines a significant destabilization in the system and leads to a breakdown of the nanomicelles, which separate in a coacervate phase in the excess of water with consequent drug release in the surrounding aqueous environment. Such a behavior is visually manifested by the formation of a cloudy/milky solution [43], whose appearance could be used as an index of successful incorporation of the drug inside the nanomicelles [27]. Nevertheless, the CP value of N2CyA-ASN formulation (45 °C) was considered suitable to provide a good stability after its instillation on the ocular surface with temperatures of 30–32 °C. A peculiar property is the regeneration time of the nanomicelles detected by cooling the formulation down up to room temperature (20 °C). In fact, micelles are generally continuously disintegrating and reforming. The RT obtained for N2CyA-ASN was 9 min, and after this time, the formulation was again optically clear. Conversely, N1CyA-ASN remained unassembled after heating, while for N3CyA-ASM, the RT occurred in a longer time (18 min). It is known that nanomicelles are in dynamic equilibrium with individual surfactant molecules that are constantly being exchanged between the bulk and the micelles. In conclusion, micellar stability and thus the micellar break-up time become the key factors in controlling technological processes that involve a rapid increase in interfacial area, such as foaming, wetting, emulsification and oil solubilization, and they can be considered a crucial point for drug release kinetics and for nanomicelle stability [44].

On the basis of these first results, the more promising nanomicellar formulation in terms of drug entrapment capacity, mean size distribution, thermal resistance and regeneration capacity appeared to be N2CyA-ASN, containing a combination of VitE-TPGS:RH-40 in a molar ratio of 1:1. Therefore, this mixture was selected to be viscosized in different conditions to produce in situ gelling systems. 

Before moving on, the critical micellar concentration (CMC) determination was performed on the selected nanomicellar mixtures to determine the minimum concentration of the surfactants required to form micelles.

**Figure 1:**
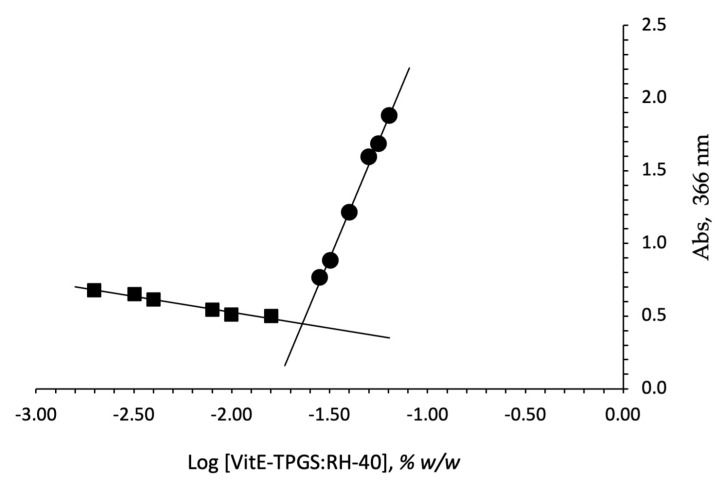
Plot of concentration of the surfactant VitE-TPGS:RH-40 (1:1 ratio) mixture vs. absorbance detected at 366 nm.

In fact, above the CMC values, the surfactants in water cause the spontaneous formation of nanomicelles. This process occurs mostly because it is thermodynamically favorable to produce a lower interfacial free energy system. The critical micellar concentration (CMC) was determined for the VitE-TPGS:RH-40 (1:1) molar ratio mixture using iodine as probe, and the obtained value was compared to that obtained by analyzing the single surfactants. Figure 1 reports the plot of absorption intensity of I_2_ (absorbance at 366 nm) as a function of the total surfactant concentration (log scale) calculated as the sum of the number of single components (VitE-TPGS plus RH-40). The CMC values were 0.026 and 0.029% *w*/*w* for VitE-TPGS and RH-40, respectively, confirming the value reported by Puig-Rigall et al. for VitE-TPGS [45] (CMC *≅* 0.02%). A lower CMC value with respect to the single surfactants, 0.023% *w*/*w*, was obtained for VitE-TPGS:RH-40 (1:1) molar ratio. The critical micelle concentration is an essential parameter influencing both in vitro stability and in vivo activity of nanomicelles. In this context, a low CMC value seems essential and, in our case, the CMC reduction to 0.023% *w*/*w* value for the VitE-TPGS:RH-40 1:1 mixture makes this formulation an ideal binary combination, showing a CMC intermediate value between those of the individual polymeric surfactants [46]. 

Finally, theoretical critical micellar concentration of the selected mixture (CMCmix), calculated by the reported formula, was compared with the experimentally determined one. The calculated theoretical CMCmix was 0.028% *w*/*w*, while the experimentally found value was 0.023% *w*/*w*. A small shift from the theoretical CMC value was observed; the lowering of the experimental CMC value for micelles based on the mixture could be considered a confirmation of the more favorable interactions between the two surfactants. A similar behavior was observed in the literature [46,47] for other typical polymeric surfactants, demonstrating how the optimization of the mixtures can be a key factor in promoting the assembly of stable nanomicelles.

### 3.2. Characterization of Fx/N2CyA-ASNg

#### 3.2.1. Size, CyA-In, CyA-EE, CyA-LE%, CP, RT, Osmolality and pH

**Figure 2:**
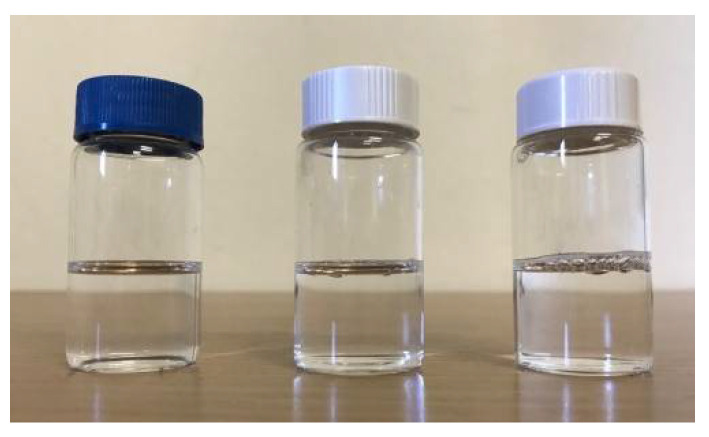
Fx/CyA-ASNg dispersions containing 0.10 (**left**), 0.15 (**center**) and 0.20% *w*/*w* (**right**) of GG-LA.

Three Fx/N2CyA-ASNg formulations based on different concentrations of gelling polymer and containing CyA loaded nanomicelles were developed with the aim to prolong the residence time in the precorneal area after ocular application. A picture of these formulations is shown in Figure 2.

The Fx/N2CyA-ASNg formulations were obtained by mixing the self-assembled nanomicelles and a polymeric dispersion enriched with optimized concentrations of monovalent and bivalent ions able to trigger the sol–gel transition of the gellan gum following the instillation. The influence of the percentage of GG-LA (0.10, 0.15 and 0.20% *w*/*w* for F1, F2 and F3 formulations, respectively) on the features of the nanomicelles was analyzed, and the results of the technological evaluation are reported in Table 4. All Fx/N2CyA-ASNg formulations showed a very small size with D_h_ (±SD), ranging from 17.03 (±0.87) to 19.72 (±1.97) nm, with a narrow PI between 0.14 ± 0.04 and 0.28 ± 0.06, indicating that the presence of gellan gum did not alter the self-assembling aptitude of the binary surfactant mixture. The amount of CyA solubilized for encapsulation into the Fx/CyA-ASNgs ranged from 0.123 to 0.146% *w*/*w*, corresponding to a high CyA-EE between 89.14 ± 3.37 and 97.28 ± 1.12% *w*/*w*. Furthermore, the results highlighted that the selected nanomicellar formulation was also able to preserve a self-assembling trend in the presence of salts and/or gelling polymer, maintaining the same technological characteristics not only in terms of encapsulation capability and size. All the Fx/N2CyA-ASNgs formulations appeared to be clear solutions at room temperature: the CP of the nanomicelles was confirmed at 45 °C, with only a slight shift to a higher RT value (10 min). In addition, the Fx/N2CyA-ASNg formulations showed osmolality and pH values in the physiological range between 304–310 mOsm/kg and 6.9–7.0, respectively. The morphological analysis confirmed that the nanomicellar formulations are able to prevent disturbing normal vision and maintain ocular transparency, representing a further important advantage for an ocular formulation.

#### 3.2.2. Gelling Capacity (GC) and Rheological Characterization

**Figure 3:**
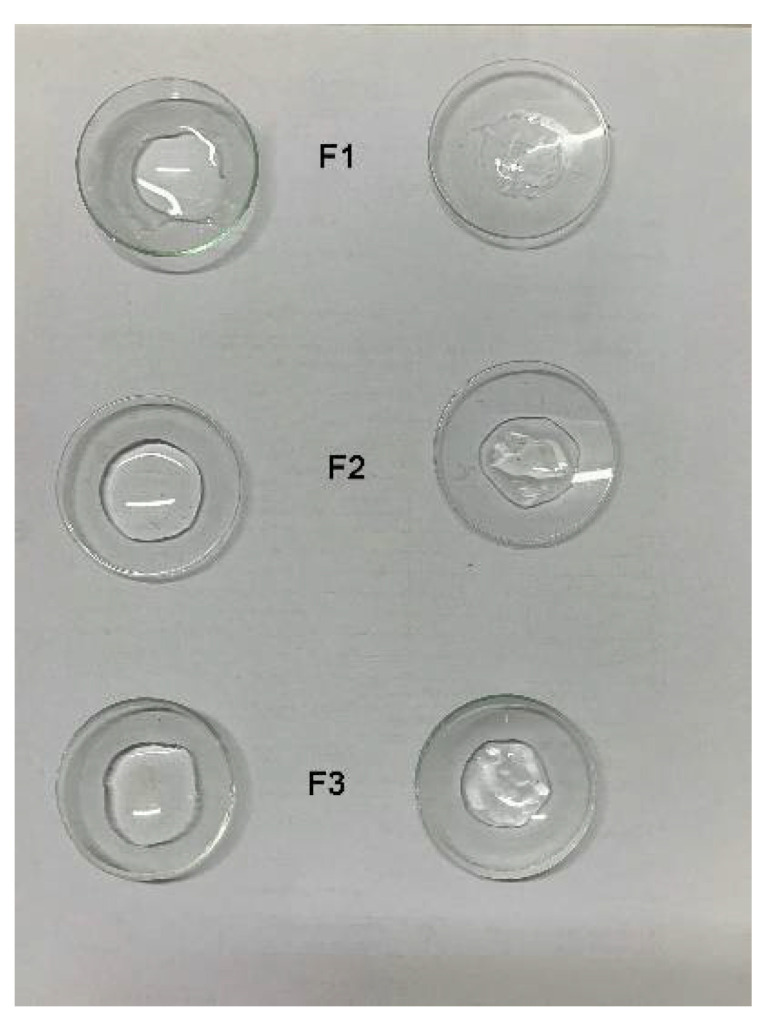
Picture of in situ gelling systems (F1–F3) containing N2 nanomicelles, Fx/CyA-ASNg (**left**) and Fx/CyA-ASNg/ATF (30:7, *v*/*v*) (**right**).

Low acetylated-gellan gum was selected as the in situ gelling system since it gives an optically clear dispersion with low viscosity after preparation and allows the formation of an ion-activation gelling system after ocular administration. Although this typical gellan gum behavior is known, the gelling capacity of systems containing different amount of gellan gum in the presence of surfactant nanomicelles (Fx/N2CyA-ASNgs formulations) is unknown. Furthermore, this research was approached for the additional purpose to identify the most suitable GG-LA concentration. In fact, providing the ocular application, rate and extent of gelation represents essential factors that can be customized as the result of the sum of both salts added in the formulation and existing in the tear fluid, as well as of the polymer concentration. The test was performed both on blank GG-LA dispersions (F1–F3) and on nanomicelle-enriched polymeric dispersions (Fx/N2CyA-ASNgs). The results highlighted that the nanomicelles did not interfere with the polymer gelling capability. It was visually observed that immediately after contact of the formulations (Fx and Fx/N2CyA-ASNgs) with artificial tear fluid, an instantaneous production (10–15 s) of a tridimensional polymeric network embedding water occurred, thanks to the right balance between divalent and monovalent cations [34]. The results are displayed in Figure 3, and the related data are summarized in Table 5. All the formulations showed immediate gelation in the time interval between 15 and 20 s depending on the different degree of gelation, according to the scale reported in Table 2. The gelling capacity essentially seems depend upon the amount of polymer added in the formulations, while no obstacles to gelation were appreciable for the presence of the nanomicelles inside gels. In all cases, significant differences were observed in the viscosity values following the phenomenon of gelling due to the addition of ATF. The best formulations in terms of GC were F2/N2CyA-ASNg and F3/N2CyA-ASNg, for which the percentage of viscosity improvement settled on 70% with respect to viscosity values before the addition of ATF, independently of the GG-LA concentration. In fact, the viscosity values increased by about 50% for F1/N2CyA-ASNg, with the lowest GG-LA concentration. 

For ocular formulations, both the dripping ability from the bottle on the ocular surface and the scarce loss from the conjunctival sac after instillation are closely related to optimal viscosity. Therefore, the development of an ocular in situ gelling system requires an optimal viscosity value, along with a rapid sol–gel transition following contact with the eye surface. 

All developed formulations showed a pseudoplastic behavior with a decrease in viscosity by increasing the shear rate in the range between 0 and 200 s^−1^. As summarized in Table 5, the viscosity values for GG-LA dispersions (Fx), calculated at low shear rate (D = 1.0 s^−1^), increased from 39.63 ± 0.23 to 136.2 ± 20.86 mPa_*_s by doubling the GG-LA concentration from 0.1 to 0.2% *w*/*w* for F1 and F3, respectively. In all cases, the dilution of the formulations with ATF in the volume ratio 30:7 (Fx:ATF), to simulate the in vivo physiological condition, determined statistically significant higher viscosity values with respect to the initial ones. In conclusion, the apparent viscosity of the Fx formulations rose with increasing polymer concentration from 0.1 to 0.2% *w*/*w* of gellan gum when in contact with ATF. In fact, formulations F2 and F3 containing 0.15 to 0.2% *w*/*w* of GG-LA showed the highest viscosity values with an improvement of about 1.8 and 3.4-fold with respect to F1 with 1% *w*/*w* of GG-LA. These increases were maintained when the formulations were mixed with ATF. This peculiar behavior of Fx/CyA-ASNg formulations could be due to the strong polymer chain interaction that tends to be closer at higher concentrations and in the presence of ATF, leading to a more structured three-dimensional network [34,48].

#### 3.2.3. Dynamic Linear Viscoelastic Range and Mechanical Spectra 

The dynamic viscoelastic experiments allowed us to determine whether the in situ gelling nanomicellar formulations were mainly in a liquid or a gel state after instillation into the eye.

An in situ gel-forming systems should undergo a phase transition to form viscoelastic gels after instillation into the conjunctival cul-de-sac of low viscosity dispersions. The changing of the viscoelastic properties, generally due to entanglement of GG-LA polymer, depends on the right balance between the appropriate content of mono- and divalent ions into the formulations and the content of salts in the tear fluid. A correct evaluation of the total salt amounts in contact with the polymeric structure immediately after instillation is essential to determine an optimal change in the viscoelastic properties and, therefore, to modify the fate of the formulations [49].

Indeed, the events occurring after instillation of an ophthalmic formulation into the precorneal area of the eye involve the dilution of the formulations due to tear fluid, the diffusion through the mucin layer, the maintenance on the corneal surface and the penetration into the cornea (epithelium, stroma and endothelium) of the CyA or the self-assembling CyA micellar systems. Then, the immediate production of an in situ gel system in the eye is important because a weak gel is expected to be produced in the eye from low viscosity dispersions.

**Figure 4:**
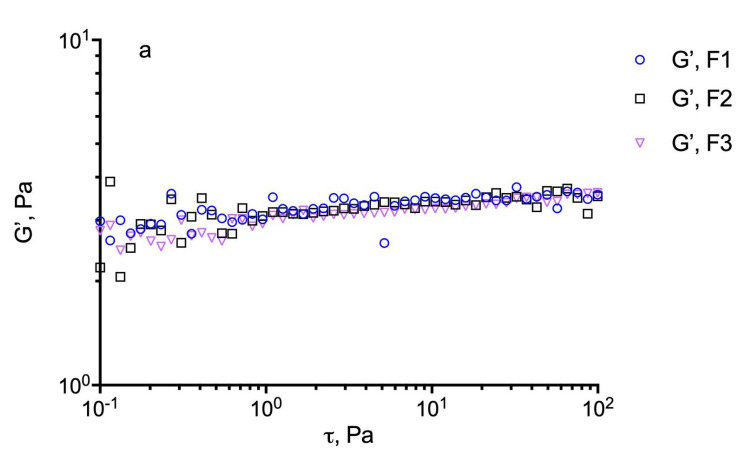

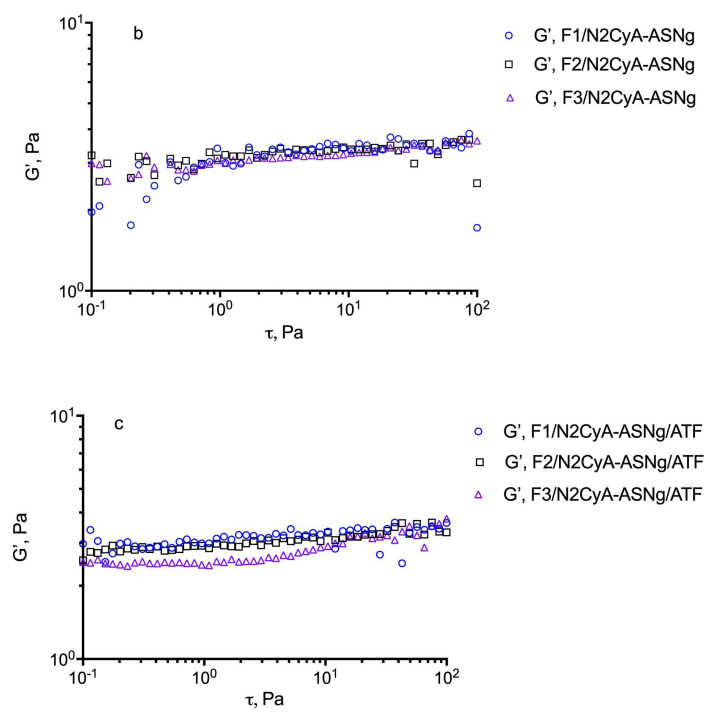
Determination of the linear viscoelastic range (DLVR). Elastic modulus (G’) at 5 Hz plotted versus shear stress (t) for: (**a**) GG-LA dispersions (Fx), (**b**) GG-LA dispersions containing nanomicelles (Fx/N2CyA-ASNg) and (**c**) GG-LA dispersions containing nanomicelles mixed with artificial tear fluid (ATF) in 30:7 ratio, (Fx/N2CyA-ASNg/ATF).

For these evaluations, an estimation of the dynamic linear viscoelastic range (DLVR) in oscillatory shear was performed by stress sweep experiments at 5 Hz. The results are illustrated in Figure 4 for the different formulations including the plain GG-LA dispersions (F1–F3) and the selected nanomicellar systems (Fx/N2CyA-ASNg) before and after (Fx/N2CyA-ASNg/ATF) the dilution with ATF (30:7 volume ratio). From this experiment, a linear viscoelastic region can be defined where G’ remained constant and the formulations were subjected to structural modifications depending on the actual condition (before and after mixing with ATF). In our case, the DLVR region was extended and quite constant for all GG-LA colloidal dispersions, regardless of polymer concentration. These data suggested that the samples were not damaged by stress, allowing the hypothesis that the formulations are highly stable at the different stress conditions that occur in vivo on the ocular surface. However, a mild reduction in dynamic linear viscoelastic region was found for F3/N2CyA-ASNg/ATF based on 0.2% *w*/*w* of + GG-LA (see Figure 4c).

**Figure 5:**
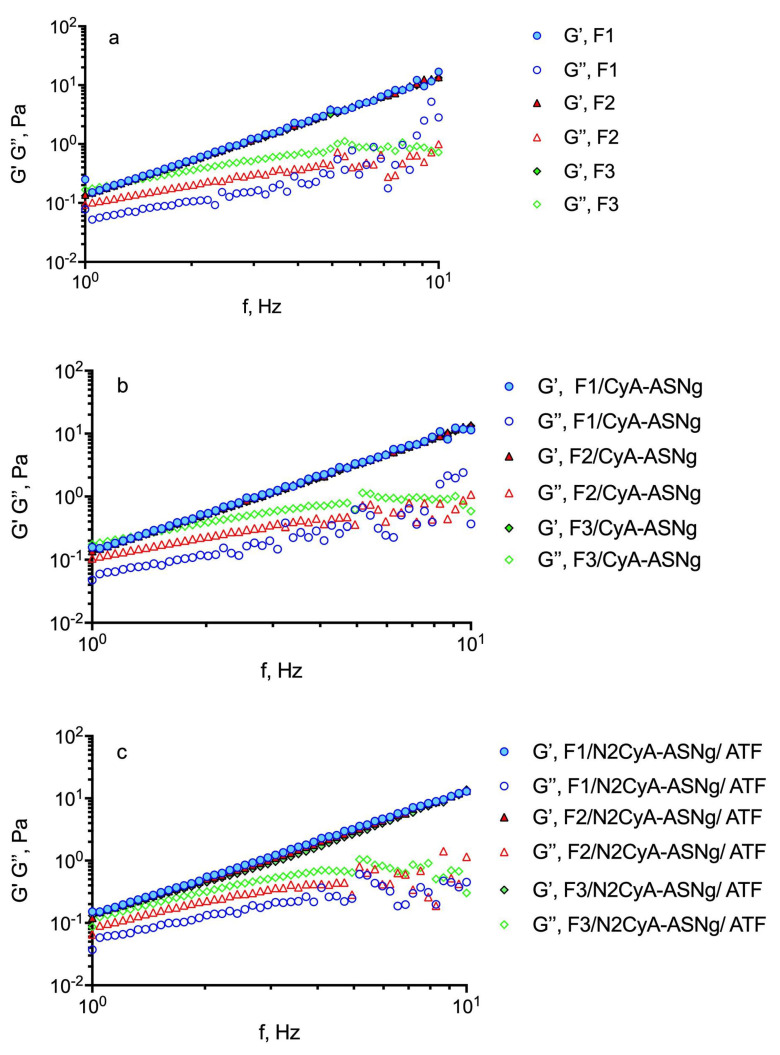
Influence of GG-LA concentration on the frequency dependence of the storage modulus (G’) (closed symbols) and the loss modulus (G’’) (open symbols) of: (**a**) GG-LA dispersions (Fx), (**b**) GG-LA dispersions containing nanomicelles (Fx/CyA-ASNg) and (**c**) GG-LA dispersions containing nanomicelles mixed with artificial tear fluid (ATF) mixed in ratio 30:7 (Fx/N2CyA-ASNg/ATF).

To determine the mechanical spectra, the frequency sweep test was carried out at a shear stress fixed at 5 Pa, based on previous experiments, i.e., within the linear viscoelastic region to avoid any sample structure damage caused by the stress set during the test. The influence of gellan gum concentration on the mechanical spectra at 32 °C in the presence of polymeric surfactant nanomicelles and after the gelation process for dilution with ATF is shown in Figure 5. In Table 6, the storage (G’) and loss (G”) moduli are reported at 1 Hz frequency. The higher the G’ values, the greater the elastic character, while the higher the G” value, the more marked the viscous properties of the preparation. All the analyzed samples exhibited an elastic modulus (G’) higher than the viscous (G’’) one throughout the whole frequency window covered and an exclusive frequency dependence for G’ that some authors found typical for gel-like materials [50]. The mechanical spectra (see Figure 5) reflect the existence of three-dimensional networks with a high degree of internal structuring, although relatively low values of elastic modulus for all the analyzed samples were detected. Moreover, since no crossover point was recorded, it can be assumed that the solid character prevails over the liquid one in aqueous GG-LA dispersions. Similar behaviors were found by Garcia Gonzalez et al. [38] for diutan and rhamsan gums, where G’ values were found to be higher than the G” values, so that the preparation exhibited an elastic-dominant behavior. Nevertheless, it is interesting to note that after dilution of the formulations with ATF (ratio 30:7), no differences in the rheological profile were observed, since the interaction of the polymer with the tear ions produced the formation of a viscous structured system that balanced the dilution effect of the tears. Moreover, it can be assumed that after instillation and following blinking of the eye lid, these pseudoplastic systems would show a decrease in viscosity that becomes partially balanced by ion activation gelling systems [51]. It is important to note that the behavior of the polymer seems not to be affected by the presence of surfactants structured in nanomicelles.

The tangent of the phase angle (tanδ), useful for establishing the presence and the extent of elasticity in a fluid, can be calculated as tanδ = G”/G’. Thus, a viscous (liquid-like) state is observed when tanδ > 1, which appeared for the higher GG-LA concentration (formulations F3). This behavior could favor a free flow from the bottle during instillation into the lacrimal sac. However, all the GG-LA concentrations are sensitive to the ions of ATF and underwent liquid/gel transition as evidenced by the reduction in tanδ values for all Fx/N2ASNg/ATFs. The behavior of gellan gum could be defined as “weak gel”, and it is considered appropriate for ocular administration.

#### 3.2.4. TEM Analysis

**Figure 6:**
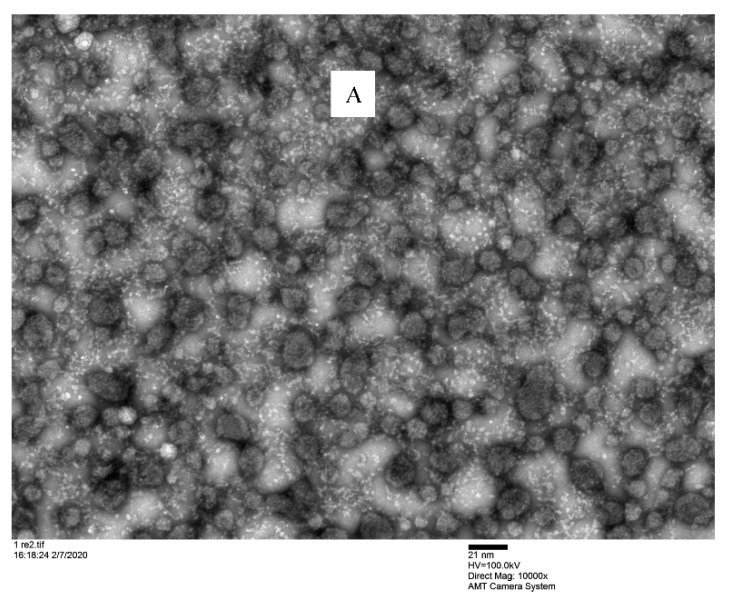

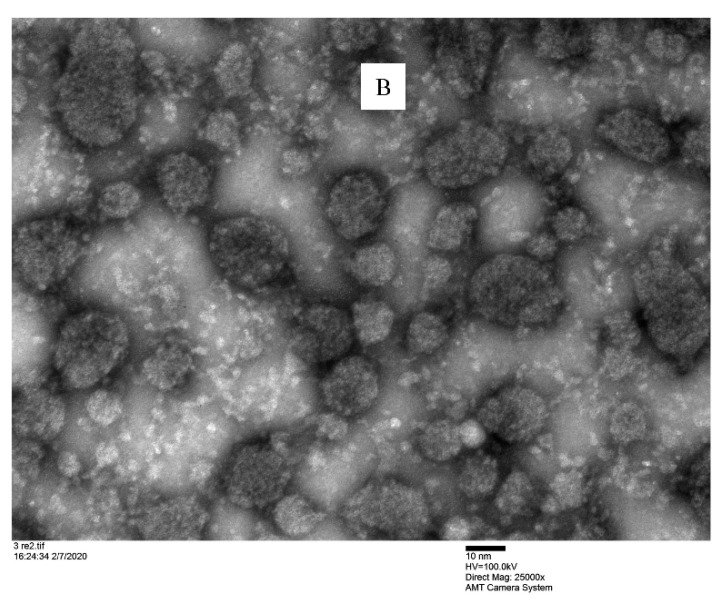
Microscopic observation of F2/N2CyA-ASNg/ATF gelled formulations under TEM. (**A**) scale bar: 21 nm; (**B**) scale bar: 10 nm.

TEM investigation showed that the nanomicelles were spherical in shape and dispersed uniformly (Figure 6) into a devoid of aggregates gelling matrix structure. This analysis confirmed that the binary mixture of surfactants (VitE-TPGS and RH-40) produced a nanomicellar structure with the hydrophobic chains involved in the inner core where the CyA is entrapped and hydrophilic terminals are able to form the hydrophilic corona. The interaction of the hydrophilic regions with the hydrated polymeric chains of gellan gum helped to maintain the uniformity. The particle sizes visualized by TEM were very similar and in agreement with the size obtained by DLS.

#### 3.2.5. Wettability of Fx/N2CyA-ASNg

Contact angle is a typical parameter useful to characterize an ophthalmic gelling formulation from a technological point of view, especially in the case of formulations subjected to structural changes as with in situ gelling systems. In fact, the prerequisite for an in situ gelling formulation is to undergo rapid transition from sol to gel under the physiological condition [52,53]. Although a complete wettability is obtained when a product wets a surface with a contact angle of 0°, angles greater than 0° up to 90° are also considered partially wettable, as required for ophthalmic application. Formulations with contact angles between 90° to 180° are partially not wettable, and greater than 180° are completely not wettable, respectively. In addition, the capability of the eye drops to spread on the ocular surface following instillation may contribute to increased ocular bioavailability. In the present work, water contact angles were measured to determine the wettability of the Fx/N2CyA-ASNg formulations before and after the gelation process (Fx/CyA-ASNg/ATF) following mixing with ATF. The selected nanomicellar formulation (N2/CyA-ASN) was characterized by a contact angle value of 33.37 ± 0.63° (see Table 7), which was lower and significantly different from the contact angle measured for MilliQ pure water, chosen as reference (44.77° ± 2.12; *p* = 0.0183, t-test). The formulations containing a lower concentration of gellan gum seemed to maintain contact angle values comparable to those of N2/CyA-ASN: 34.27 ± 1.74° and 34.03 ± 2.03°, respectively, for F1/N2CyA-ASNg (0.1% *w*/*w*) and F2/N2CyA-ASNg (0.15% *w*/*w*). A mild improvement was measured for F3/CyA-ASNg gelling formulation containing the highest GG-LA concentration (0.2% *w*/*w*), but this contact angle value was not statistically different from the others and from N2/CyA-ASN nanomicellar preparation. Furthermore, similar contact angle values were detected after mixing the in situ gelling preparations with simulated tear fluid, demonstrating that the increased entanglement of GG-LA polymer chains due to the presence of ion concentration did not cause any changes in this parameter. The high wettability of the viscosized nanomicellar formulations before and after dilution with ATF was probably due to the presence of the surfactant nanomicelles. Indeed, literature data demonstrate that gellan gum dispersions do not show any significant change in air–water and oil–water interfacial tensions.

These findings suggest that the Fx/N2CyA-ASNg formulations could more easily wet and adhere to the surface of the cornea than the aqueous vehicle with a spreading capacity on the cornea more energetically favored than non-spreading. Low contact angle values are required for an ophthalmic product as they are associated with a greater ability of the product to spread out over the surface of the eye by improving mixing with the tear film and sometimes increasing its stability. Similar data were detected for other viscous ophthalmic formulations containing hyaluronic acid, while lower contact angles were measured for other types of formulations, such as nanoemulsions, with a higher content of surfactant [9].

#### 3.2.6. Storage Stability

The development of nanomicellar formulations for ocular application is subject to the maintenance of their technological characteristics. For this aim, the ability of Fx/N2CyA-ASNg systems to maintain both physical and chemical stability, including the CyA cargo load into the micellar structure, was monitored over time up to 60 days by storing the formulations at 4 and 20 °C. The physical parameters, such as size (D_h_) and PI, were evaluated by light scattering analysis; the amount of CyA remaining in the formulations (CyA-EE) was detected by HPLC, and their appearance was visually checked to identify any trace of CyA precipitation phenomena. 

In all cases, the formulations remained clear, without precipitation of the drug. As shown in Table 8, the physical parameters and the encapsulated CyA in the nanomicellar structure for the different gelling systems before and after storage at 4 and 20 °C were considerably comparable. Furthermore, the CV% was higher for the storage at 20 °C, but remained lower than 5%, meeting the acceptance criteria of international guidelines (Eudralex 3AQ11a; Guideline: Specifications and Control Tests on the Finished Product—75/318/EEC as amended; EMA (2015) Guideline on Bioanalytical Method Validation, European Medicine Agency, London, UK).

These results demonstrated no significant change in stability for a period of 60 days. Nevertheless, a minimal variation in the D_h_ values was observed, and no alterations in physical appearance were found in the different in situ gelling systems, which in any case remained in the required dimensional range. 

#### 3.2.7. Cytotoxicity Assay

**Figure 7:**
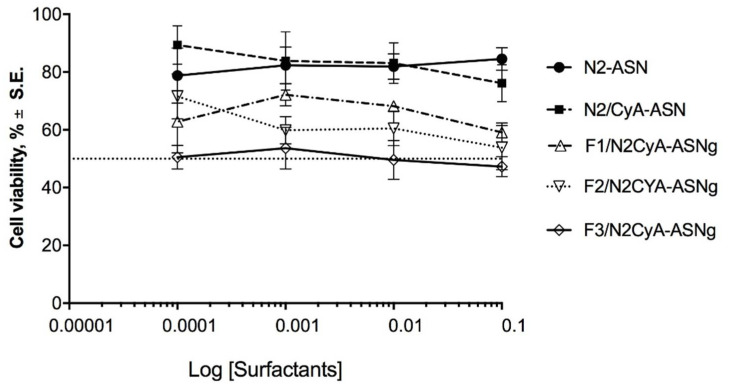
Cytotoxicity of surfactant self-assembling nanomicellar preparations with (N2CyA-ASN) and without (N2ASN) encapsulated CyA and surfactant self-assembling nanomicellar preparations in in situ gelling systems based on different concentrations of GG-LA (Fx/CyA-ASNg) formulations on rabbit corneal epithelial cell line.

The results of the cytotoxicity test are shown in Figure 7, where the percentage of viable cells is reported as a function of the concentration of surfactants, an essential part of the prepared nanomicelles and a critical component in terms of cytotoxicity. This choice was also made considering that, in a previous study, the same authors [27] evaluated the cytotoxicity of a CyA solution obtained by diluting a DMSO stock CyA solution, which seemed to maintain cell viability of around 75%, except for the highest concentration, which determined a reduction in viability to 58%. On the basis of this information, in the current work, we focused our attention on the influence of the surfactants towards cell viability. The treatment of the cell line with the different preparations did not reveal a remarkable toxic effect. Cell viability was above 50% for all tested concentrations, with the exception of F3/N2CyA-ASNg formulation, which fluctuated in the acceptable 50% cell viability limit for all the tested concentrations (Figure 7). Both blank nanomicelles (N2-ASN) and those containing drug (N2/CyA-ASN) maintained cell viability above 80% with no statistically significant difference between the two formulations. The addition of GG-LA (F1-F3/N2CyA-ASNg) produced a decrease in cell viability up to 50% for the highest polymer concentration. This behavior appeared to be closely related to the GG-LA concentration and consequently to the degree of gelling. 

#### 3.2.8. In Vitro Permeation Studies of CyA through Ocular Reconstituted Tissue

EpiCorneal^TM^ tissue, a three-dimensional corneal tissue model derived from human corneal epithelium, was used to study the CyA permeation through corneal epithelium from an encapsulated drug nanomicellar system. It represents the apical surface of the epithelium, a significant barrier for the permeability of ophthalmic drugs through the cornea and contains tight-junctions and desmosomes, expressing tight junction proteins in the apical cell layers developing TEER above 1000 Ohm/cm^2^. The in vitro permeation studies of CyA using EpiCorneal™ tissue were performed on the nanomicellar formulation N2CyA-ASN using Ikervis^®^ emulsion, as reference [27]. Even for this series of experiments, the CyA permeation through the Epicorneal™ tissues model was not observed, and taking into account the low sensibility of the HPLC method corresponding to (LOQ was 50 ng/mL), any CyA permeation can be excluded. These results are analogous to the previously obtained CyA encapsulated nanomicelles that were a similar nanomicellar ophthalmic system for size and composition. Nevertheless, the small dimension nanomicelles based on neutral surfactants seemed not to favor the permeation through the main barrier of the cornea represented by the epithelium.

At the end of the drug permeability experiments (after 4 h of incubation), changes in 3D corneal tissue barrier integrity were again evaluated by measuring TEER. The reduction in the resistance of Epicorneal™ tissue treated with N2/CyA-ASN formulation with respect to baseline data was of 73.21 ± 1.96% (S.E., *n* = 6), a value that tends to be higher than that obtained by Ikervis^®^ (65.0 ± 6.44%, S.E., *n* = 6), although there are no statistically significant differences. The nanomicellar formulation has good biocompatibility with the ocular tissue, as demonstrated by these data. The same research group has already reported a permeation study through Epicorneal™ tissue, demonstrating that the presence of an irritating substance, e.g., benzalkonium chloride, in the eye drop could significantly decrease the TEER values at the end of the experiment by up to 50% [27].

#### 3.2.9. In Vivo Pharmacokinetic Studies: CyA Content in Tear Fluid of Rabbits

To demonstrate that the nanomicelles were able to deliver CyA into ocular tissues by using the proposed nanomicellar technology associated with ion activated gelling systems, healthy rabbit eyes were selected for a precorneal pharmacokinetic study. Here, the optimized CyA nanomicellar formulation was added to different GG-LA concentrations in an aqueous vehicle where an equilibrium among mono- and divalent ions was chosen. According to Majeed and Khan [25], gelation should prolong the residence time of the drug at the target site and should increase its bioavailability, calculated as the area under curve.

**Figure 8:**
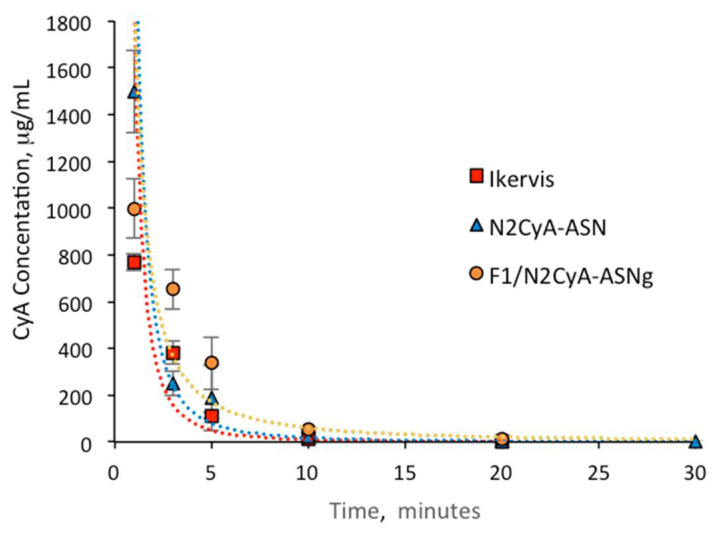
In vivo pharmacokinetics profile of Ikervis^®^, N2/CyA-ASN and F1/N2CyA-ASNg (*n* = 6, mean ± SE).

**Figure 9:**
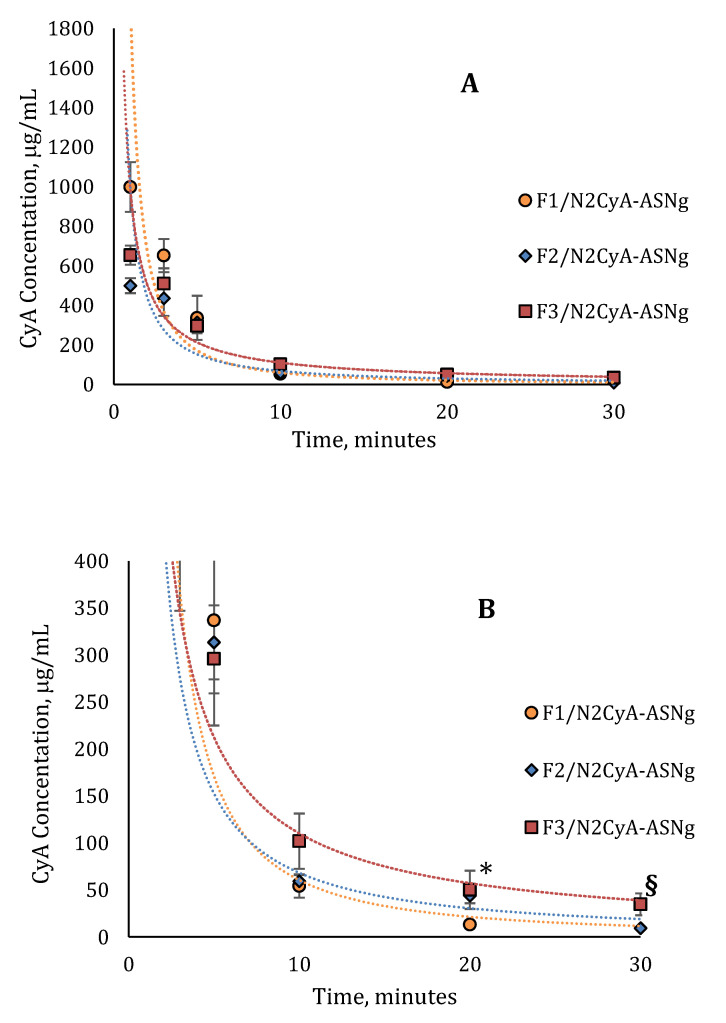
In vivo pharmacokinetics profile of (**A**) nanomicellar fomulations with different concentrations of GG-LA (*n* = 6, mean ± SE); (**B**) magnification of pharmacokinetics profile from 0 to 400 μg/mL. * Statistically different from F1/N2CyA-ASNg; § statistically different from F2/N2CyA-ASNg.

The pharmacokinetic profiles of the in situ gelling nanomicellar preparations were compared with commercial CyA eye drops of the same concentration of drug (Ikervis^®^, 0.1%) following the same procedure of biological sample collection. The results are shown in Figure 8 and Figure 9, and the main pharmacokinetic parameters, calculated by assuming first order pharmacokinetics profiles for the concentration of CyA into tear fluid vs. time, are summarized in Table 9.

The CyA concentration in the tear fluid of rabbit eyes treated with the micellar formulation was significantly higher immediately after administration (C_1min_ for N2CyA-ASN = 1497.72 ± 174.8 μg/mL) in comparison to Ikervis^®^ and to the experimental products with higher GG-LA concentration (F2-F3/N2CyA-ASNg) (*p* < 0.05, two tails unpaired t-test). Nevertheless, the nanomicellar formulation produced a quick elimination of drug from tear fluid, as graphically shown in Figure 8 and Figure 9, with a concentration of CyA in tear fluid of 251.12 μg/mL just 3 min after instillation. The elimination rate constant for the N2CyA-ASN nanomicellar system was reduced more than two times if compared to that calculated for Ikervis^®^ (K_e_ = 0.091 and 0.205 min^−1^ for N2CyA-ASN and Ikervis^®^, respectively), causing less rapid CyA elimination form the eye. In conclusion, the nanomicellar formulation increased the CyA bioavailability by approximately 2-fold if calculated between 1 and 30 min, but only by 1.2-fold with respect to commercial reference if the 3–30 min interval was considered to determine the AUC.

A significant increase in the CyA concentration was obtained when nanomicelles were formulated in an in situ gelling system. Although the CyA concentration one minute after instillation was considerably low (varying from 0.1 to 0.2% *w*/*w* of GG-LA in the formulations F1–F3), the gelling systems maintained higher CyA concentrations up to 30 min with an increase in bioavailability, calculated as the area under curve tear fluid CyA concentration vs. time, of more than 2-fold with respect to Ikervis^®^ (AUC_1min_ = 3953, 2127 and 1813 μg/mL min^−1^ for F1/N2CyA-ASN, N2CyA-ASN and Ikervis^®^, respectively) and of about 6-fold compared to N2CyA-ASN (AUC_3min_ = 2982 and 500.7 μg/mL min^−1^ for F3/N2CyA-ASN and N2CyA-ASN, respectively).

Immediately after instillation, the addition of gellan gum did not provide a positive effect on the CyA concentration, but rather a reduction in the drug concentration in tear fluid, already detected with GG-LA 0.1% *w/w* (C_1min_ = 998.37 ± 126.17 μg/mL for F1/N2CyA-ASN). This behavior could essentially depend on the gelling phenomena that obstacle the dilution of the instilled drop when the GG-LA-based formulations were applied, determining the possibility that a “gel reservoir” remains in the cul-de-sac of rabbit eyes. In fact, during the pharmacokinetic test, only the liquid component of the lacrimal fluid was collected with the microcapillary tube, while the formed gelling system remained a stable viscous formulation in the conjunctival sac. Therefore, the gelling system stayed in the eye for longer, and then it was gradually mixed and diluted with the tears.

Such behavior is proven by pharmacokinetic parameters calculated for the in situ gelling systems, and it can explain the differences in the elimination rate constant and consequently the values obtained for the t_1/2_. 

The application of the nanotechnology to design an ocular drug delivery system able to favor the maintenance of high drug amounts to target sites has been planned by several researchers, but in this study, we designed an in situ gel incorporating nanomicelles to ensure a prolonged retention time into the precorneal area where CyA could exert its peculiar pharmacological activity. The combination of nanotechnologies, such as nanoemulsion [54], nanoparticles [55,56] and micelles [51], with in situ gelling systems was applied by several researchers. In particular, Nagai et al. [53] studied a combination of nanoparticles in a gelling system based on methylcellulose. Nevertheless, the size of the nanoparticles and their compositions were different with respect to our formulations. Moreover, a prolonged pre-corneal and pre-conjunctival contact time of the drug was demonstrated in comparison with commercial eye drops by an optimization of the concentration of gelling polymer.

## 4. Conclusions

The present data demonstrate the potential of VitE-TPGS/RH-40 polymeric micelles in ocular drug delivery for their ability to improve CyA solubility and to enhance its residence time in tear fluid, above all when instilled as an in situ gelling system.

VitE-TPGS is a widely used commercial adjuvant in drug delivery; as a potent P-gp inhibitor, it has been used by several authors both to promote the interaction with the ocular structures allowing the drug transport through the corneal epithelium and to maintain a high drug concentration at the ocular level. Several diseases of the surface of the eye, such as dry eye disease, could take advantage of the presence of a high drug concentration in the epithelium. 

Surfactant nanomicelles, in addition to technological advantages such as optimal size for ocular application and high capacity of drug encapsulation, can be strengthened in their effectiveness by associating them with in situ gelling systems. In particular, starting from the knowledge of the great impact of gelation phenomena on ocular bioavailability, we optimized the formulations both from qualitative and quantitative points of view, selecting the concentration of GG-LA and the percentage of mono- and divalent ions able to promote the gelation of the instilled eye drops.

These new formulations, while providing the same ease of instillation as conventional eye drops, appear to be a valid alternative in ophthalmic therapy due to their ability to enhance drug residence time in the precorneal area, representing a useful innovative ODDS for the treatment of several affections of the anterior segment of the eye. 

## Figures and Tables

**Table 1:** Composition of Cyclosporine-A loaded nanomicellar formulations (NxCyA-ASN).

Formulations	VitE-TPGS:RH-40	VitE-TPGS	RH-40
	Molar Ratio	(% *w*/*w*)	(% *w*/*w*)
N1CyA-ASN	2.25:1	1.35	1.0
N2CyA-ASN	1:1	0.6	1.0
N3CyA-ASN	0.5:1	0.3	1.0

**Table 2:** Characterization of the grade of gelation.

Degree of Gelation	Grade
No gelation	-
Weak gelation, gel dissolves rapidly	+
Immediate gelation with less stiff gel formation that remain for a few hours	++
Immediate gelation with stiff gel formation that remain for an extended period of time	+++
Very stiff gel	++++

**Table 3:** Size (D_h_), polydispersity index (PI), CyA-In, CyA-EE, CyA-LE, cloud point (CP) and regeneration time (RT) of NxCyA-ASN, (mean, *n* = 3).

Formulations	D_h_ nm ± S.D.	PI ± S.D.	CyA-In% *w*/*w* ± S.D.	CyA-EE% *w*/*w* ± S.D.	CyA-LE% *w*/*w* ± S.D.	CP °C	RT min
N1CyA-ASN	15.7 ± 0.56	0.26 ± 0.06	0.131 ± 0.006	92.83 ± 0.83	5.12 ± 0.25	>80	-
N2CyA-ASN	15.8 ± 0.26	0.16 ± 0.02	0.144 ± 0.003	99.07 ± 1.61	8.22 ± 0.14	45	9
N3CyA-ASN	16.9 ± 0.28	0.38 ± 0.03	0.063 ± 0.06	55.86 ± 2.21	2.55 ± 0.10	40	18

**Table 4:** Size, polydispersity index (PI), CyA-In, EE, LE, cloud point (CP) and regeneration time (RT) of developed Fx/N2CyA-ASNg (mean, *n* = 3).

Formulation	Size, nm ± S.D.	PI ± S.D.	CyA-In% *w/w* ± S.D.	CyA-EE% *w/w* ± S.D.	CyA-LE% *w/w* ± S.D.	CP °C	RT min
F1/N2CyA-ASNg	19.72 ± 1.97	0.14 ± 0.04	0.146 ± 0.06	97.28 ± 1.12	8.08 ± 0.10	45	10
F2/N2CyA-ASNg	17.03 ± 0.87	0.23 ± 0.06	0.143 ± 0.08	95.28 ± 5.71	7.98 ± 0.47	45	10
F3/N2CyA-ASNg	19.40 ± 1.52	0.28 ± 0.06	0.123 ± 0.05	89.14 ± 3.37	7.41 ± 0.28	45	10

**Table 5:** Rheological parameters of GG-LA formulations before and after mixing with ATF (30:7 volume ratio) (mean ± SD, *n* = 3).

Formulations	Degree of Gelation	Viscosity mPa s
F1/N2CyA-ASNg	+	39.63 ± 0.23
F1/N2CyA-ASNg/ATF	+	60.75 ± 2.08 *
F2/N2CyA-ASNg	++	70.34 ± 5.19
F2/N2CyA-ASNg/ATF	++	119.6 ± 3.39 *
F3/N2CyA-ASNg	+++	136.2 ± 20.86
F3/N2CyA-ASNg/ATF	+++	210.1 ± 14.71 *

* Significantly different from the same formulation before mixing with ATF.

**Table 6:** Values of G’, G’’ and tanδ for formulations before and after gelation in the presence of simulated tear fluid of under study formulations (mean ± SD. *n* = 3).

Formulation. Fx	Storage Modulus. G’ (Pa)	Loss Modulus G’’ (Pa)	Tangent of the Phase Angle. tanδ
F1	0.249 ± 0.030	0.078 ± 0.018	0.320 ± 0.112
F2	0.138 ± 0.010	0.093 ± 0.014	0.677 ± 0.094
F3	0.133 ± 0.002	0.167 ± 0.015	1.263 ± 0.127
F1/CyA-ASNg	0.158 ± 0.034	0.048 ± 0.006	0.319 ± 0.120
F2/CyA-ASNg	0.137 ± 0.006	0.105 ± 0.003	0.768 ± 0.017
F3/CyA-ASNg	0.136 ± 0.001	0.175 ± 0.009	1.290 ± 0.066
F1/CyA-ASNg/ATF	0.152 ± 0.025	0.037 ± 0.016	0.250 ± 0.056
F2/CyA-ASNg/ATF	0.121 ± 0.020	0.065 ± 0.021	0.438 ± 0.061
F3/CyA-ASNg/ATF	0.132 ± 0.030	0.087 ± 0.009	0.674 ± 0.096

**Table 7:** Contact angle measurements (mean ± SD, *n* = 6).

Formulations	Contact Angle before Gelation (°)	Formulations	Contact Angle after Gelation (°)
N2/CyA-ASN	33.37 ± 0.63	-	-
F1/N2CyA-ASNg	34.27 ± 1.74	F1/N2CyA-ASNg/ATF	35.90 ± 3.78
F2/N2CyA-ASNg	34.03 ± 2.03	F2/N2CyA-ASNg/ATF	35.73 ± 3.53
F3/N2CyA-ASNg	36.66 ± 1.73	F3/N2CyA-ASNg/ATF	34.24 ± 2.12

**Table 8:** Storage stability of Fx/CyA-ASNg at (**A**) 4 °C and (**B**) room temperature over 60 days (mean ± SD, *n* = 3).

(A)			
Formulations	D_h_ nm	PI	CyA-EE% *w*/*w*
F1/N2CyA-ASNg	16.42 ± 0.35	0.24 ± 0.09	91.69 ± 5.71
F2/N2CyA-ASNg	16.90 ± 0.35	0.25 ± 0.08	93.00 ± 2.78
F3/N2CyA-ASNg	18.05 ± 0.45	0.32 ± 0.07	85.52 ± 3.71
(**B**)			
**Formulations**	**D_h_ nm**	**PI**	**CyA-EE% *w*/*w***
F1/N2CyA-ASNg	16.20 ± 0.26	0.16 ± 0.08	94.28 ± 5.27
F2/N2CyA-ASNg	17.07 ± 0.29	0.31 ± 0.04	89.20 ± 3.27
F3/N2CyA-ASNg	17.80 ± 0.35	0.17 ± 0.09	86.76 ± 8.09

**Table 9:** In vivo pharmacokinetics parameters of CyA in rabbit tear fluid (mean ± SE, *n* = 6).

Formulations	C_1min_ (μg mL^−1^)	AUC_1min_ (μg/mL min^−1^)	AUC_3min_ (μg/mL min^−1^)	K_e_ (min^−1^)	t_½_ (min)
Ikervis^®^ *	769.16 ± 43.50	1813 ± 354.1	868.3 ± 128.4	0.205 ± 0.033	3.83 ± 0.74
N2CyA-ASN	1497.72 ± 174.8	2127 ± 87.82	500.7 ± 125.3	0.091 ± 0.010	7.97 ± 0.79
F1/N2CyA-ASN	998.37 ± 126.17	3953 ± 756.8	2302 ± 548.4	0.097 ± 0.009	7.48 ± 0.75
F2/N2CyA-ASN	499.31 ± 27.63	--	2459 ± 290.1	0.065 ± 0.007	11.48 ± 1.39
F3/N2CyA-ASN	653.93 ± 48.63	--	2964 ± 415.4	0.044 ± 0.004	16.61 ± 1.81

* From [27].

## Data Availability

Not applicable.
